# Applying the National Genomic DNA Reference Materials to Evaluate the Performance of Nanopore Sequencing in Identifying Thalassemia Variants

**DOI:** 10.1002/jcla.70044

**Published:** 2025-05-20

**Authors:** Xingyu Wei, Xu Yang, Wanqing Han, Li Zhang, Guojun Ouyang, Shoufang Qu, Fang Yang, Xuexi Yang

**Affiliations:** ^1^ Department of Fetal Medicine and Prenatal Diagnosis Zhujiang Hospital, Southern Medical University Guangzhou People's Republic of China; ^2^ Institute of Antibody Engineering School of Laboratory Medicine and Biotechnology, Southern Medical University Guangzhou People's Republic of China; ^3^ Guangzhou Darui Biotechnology Co. Ltd. Guangzhou People's Republic of China; ^4^ National Institutes for Food and Drug Control Beijing People's Republic of China

**Keywords:** Long read sequencing, nanopore sequencing, national reference material, thalassemia, variant

## Abstract

**Objectives:**

Nanopore sequencing shows advantages in detecting single nucleotide variations (SNVs), deletions, and complex structural variants as a single test in thalassemia. However, the performance evaluation or verification of this method remains unestablished, which is essential before clinical utility and panel registration. Here, we developed a classification method for thalassemia mutations, enabling automated interpretation, visual representation, and identification of diverse mutation types.

**Methods:**

We used a total of 36 samples, comprising 32 reference materials and four clinical samples to assess the performance of nanopore sequencing in identifying variants in terms of concordance, precision, and the lower limits of detection.

**Results:**

Our analysis successfully identified 19 SNVs, six deletions, and two triplications using nanopore sequencing across all samples. Notably, these variants showed complete concordance of 100% with the genotypes of the reference materials and known results. The precision of nanopore sequencing for detecting thalassemia variants was consistently high, with neither false positive nor false negative observed. Furthermore, the lower limits of detection achieved in our study were 3 ng/μL.

**Conclusions:**

Overall, our study proved that the reference materials can be used to evaluate the performance of nanopore sequencing in identifying thalassemia mutations, and it is necessary to incorporate triplications when utilizing reference materials for performance evaluation of long‐read sequencing. The consistent and robust performance of nanopore sequencing in this study demonstrates its potential as a reliable method for comprehensive variant detection in thalassemia and other genetic diseases diagnosis.

## Graphical abstract

This study mainly to evaluate the performance of nanopore technology in thalassemia detection by utilizing the national genomic reference materials in China. We proved that the reference materials can be used to evaluate the performance of nanopore sequencing in identifying thalassemia mutations, and it's necessary to incorporate triplicates when utilizing reference materials for performance evaluation of long‐read sequencing.

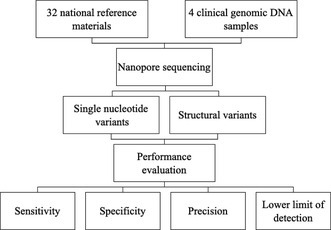

## 1 Introduction

The thalassemia, characterized by abnormal protein synthesis leading to reduced or even absent α and/or β globin chains, is the most common human monogenic disease worldwide [1, 2, 3]. According to the underlying genetic mutation and affected globin‐chain subunits within the hemoglobin tetramer, thalassemia can be broadly categorized into α‐thalassemia and β‐thalassemia.

The molecular diagnosis of various types of thalassemia is particularly challenging mainly due to the complex and multiplex nature of mutations in the *HBA* and *HBB* genes. The globin genes are GC‐rich and highly homologous, such as the two nearly identical genes in the α‐globin region, *HBA1* and *HBA2*, and two in the β‐globin region, *HBB* and *HBBP1*. Furthermore, a total of 1865 hemoglobin genetic variants have thus far been identified (https://globin.bx.psu.edu/), among which 537 mutations are thalassemia mutations [4]. α‐thalassemia variants consist of deletions, single nucleotide variations (SNVs) and other special *HBA* structural variations (SVs), including the triplicated α‐globin genes (ααα^anti3.7^ and ααα^anti4.2^) and fusion allele (*HKαα*) [5]. β‐thalassemia is caused by mutations resulting from SNVs, small deletions or insertions, and in rare cases, gross deletions [3].

Routine diagnosis and screening methods of thalassemia include gap‐polymerase chain reaction (GAP‐PCR) and the reverse dot blot hybridization (RDB). These methods are utilized for the detection of common deletions/SNVs. However, the detection of rare or novel deletions/SNVs requires multiple ligation dependent probe amplification (MLPA) and sanger sequencing. While next‐generation sequencing (NGS) permits multiplex and high‐throughput detection of genetic variants, such technology is constrained by short reads and high false‐positive rates [6]. Given the intricate nature of thalassemia mutations and the clinical asymptomatic status of most carriers, detecting novel and rare mutations such as α‐globin triplications and large deletions [7, 8] by traditional technologies is challenging, often leading to misdiagnosis and missed diagnosis. Additionally, the triplication of α‐globin genes can interact with β‐thalassemia mutations, resulting in more severe phenotypes [2, 9, 10]. Hence, improved methods for the molecular diagnosis of thalassemia are clinically imperative.

Long‐read third generation sequencing (TGS) has the potential to analyze the globin genes in single reads and directly detect rare and clinically relevant variants, including SNVs, copy number variants (CNVs), and complex SVs [11, 12, 13], which are difficult to identify by traditional thalassemia genetic testing. Recently, this technology—utilizing either the Pacific Biosciences (PacBio) or Oxford Nanopore Technology (ONT) platform—has been validated in diagnosing and carrier testing of thalassemia with a wide detection range and high accuracy [5, 7, 14, 15, 16, 17, 18]. These new technologies exhibit significant advantages in detecting SVs, particularly in homologous regions and complex SVs. Compared with PacBio, nanopore sequencing is generally more portable and affordable, making it accessible to a wide range of researchers and laboratories [19].

Before introducing for patient testing, analytic performance establishment or verification is required for a newly developed molecular genetic testing. Regulatory and accreditation requirements of clinical genetic testing laboratories recommend using reference materials (RMs) in assay development and quality assurance according to professional practice guidelines [20, 21, 22]. In 2017, the National Institutes for Food and Drug Control in China developed a genomic DNA RM panel for thalassemia genetic testing (Table 1), which covers 25 different variations with 29 distinct genotypes. This genomic DNA RM panel contains common and rare variations that represent the vast majority of thalassemia carriers in China [23]. However, this national reference did not include triplications due to the limitations of traditional methods in identifying such complex SVs. It is noteworthy that there were no prior performance evaluations of nanopore sequencing in thalassemia variant identification before this study. Here we aim to comprehensively evaluate the performance of nanopore sequencing identifying thalassemia mutations using the RMs in China and triplication samples.

**TABLE 1:** The hemoglobin genotypes of RMs and clinical samples and the performance evaluation metrics and corresponding selected samples.

Sample resource	Samples name	Genotype	Variant type	Performance evaluation	
				Precision (×10)	LOD
National genomic reference	YSH2015‐0000	Wild type			
	YSH2015‐0001	Wild type			√
	YSH2015‐0002	Hb E/β^N^	*HBB*: c.79G>A	√	
	YSH2015‐0003	−28 (A>G)/β^N^	*HBB*: c.−78A>G		
	YSH2015‐0004	IVS‐II‐654 (C>T)/β^N^ ‐‐^SEA^/αα	*HBB*: c.316‐197C>T ‐‐^SEA^		√
	YSH2015‐0005	‐‐^SEA^/αα	‐‐^SEA^		
	YSH2015‐0006	−α^3.7^/‐‐^SEA^	−α^3.7^ ‐‐^SEA^		
	YSH2015‐0007	−α^3.7^/αα	−α^3.7^	√	
	YSH2015‐0008	IVS‐I‐1 (G>T)/β^N^	*HBB*: c.92+1G>T		
	YSH2015‐0009	Wild type			
	YSH2015‐0010	Codons 71/72 (+A)/β^N^	*HBB*: c.216_217insA/T		
	YSH2015‐0011	Codon 17 (A>T)/β^N^	*HBB*: c.52A>T		√
	YSH2015‐0012	α^QS^ α/αα	*HBA2*: c.369C>G	√	
	YSH2015‐0013	5′UTR; +43 to +40 (−AAAC)/β^N^	*HBB*: c.‐11_−8delAAAC		
	YSH2015‐0014	Codon 43 (G>T)/β^N^	*HBB*: c.130G>T		
	YSH2015‐0015	Initiation Codon (ATG>AGG)/β^N^	*HBB*: c.2T>G	√	√
	YSH2015‐0017	Codon 37 (G>A)/β^N^	*HBB*: c.113G>A		
	YSH2015‐0018	Codons 41/42 (−TTCT)/β^N^	*HBB*: c.126_129delCTTT		
	YSH2015‐0019	−α^4.2^/αα	−α^4.2^		√
	YSH2015‐0020	Codons 27/28 (+C)/β^N^	*HBB*: c.84_85insC		
	YSH2015‐0021	α^CS^α/αα	*HBA2*: c.427 T>C		
	YSH2015‐0022	α^WS^α/αα	*HBA2*: c.369C>G		
	YSH2015‐0023	−50 G>A/β^N^	*HBB*: c.−100G>A		√
	YSH2015‐0024	−29 (A>G)/β^N^	*HBB*: c.−79A>G		
	YSH2015‐0026	Gγ(Aγδβ)^0^/β^N^	Gγ(Aγδβ)^0^	√	
	YSH2015‐0027	Codons 41/42 (−TTCT)/Gγ(Aγδβ)^0^	*HBB*: c.126_129delCTTT Gγ(Aγδβ)^0^		
	YSH2015‐0028	Codons 14/15 (+G)/β^N^	*HBB*: c.45_46insG		
	YSH2015‐0029	−90 (C>T)/β^N^	*HBB*: c.‐140C>T		
	YSH2015‐0030	−α^4.2^/−α^4.2^ IVS‐II‐654 (C>T)/β^N^	−α^4.2^ *HBB*: c.316‐197C>T		√
	YSH2015‐0031	IVS‐II‐654(C>T)/IVS‐II‐654 (C>T) ‐‐^SEA^/αα	*HBB*: c.316‐197C>T ‐‐^SEA^		
	YSH2015‐0032	SEA‐HPFH/β^N^	SEA‐HPFH	√	
	YSH2015‐0033	‐‐^THAI^/αα	‐‐^THAI^		
Clinical sample	S1	ααα^anti3.7^/αα	ααα^anti3.7^	√	
	S2	ααα^anti3.7^/αα	ααα^anti3.7^		
	S3	ααα^anti4.2^/αα	ααα^anti4.2^		√
	S4	ααα^anti4.2^/αα	ααα^anti4.2^	√	

## 2 Materials and Method

### 2.1 Sample

**FIGURE 1:**
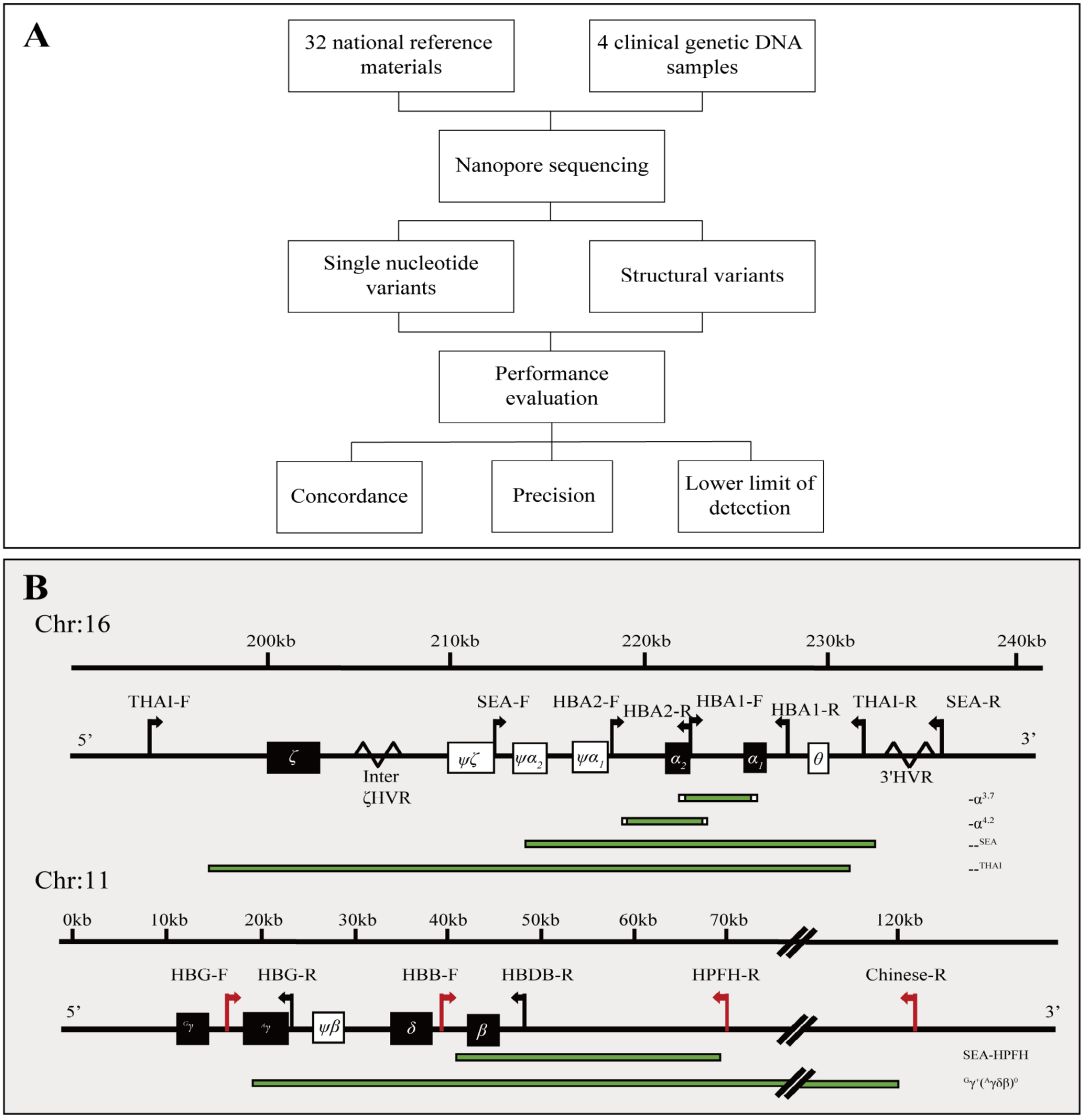
The selection diagram and amplicons for performance evaluation. (A) The selected diagram of samples for performance evaluation. (B) Primer positions and target amplicon ranges of multiplex PCR for α−/β‐globin genes.

To evaluate the performance and clinical utility of our assay for confirming thalassemia diagnosis, we obtained 32 DNA samples from the RMs for thalassemia in China (360014–201701) and four samples with α‐globin triplications, comprising two cases of ααα^anti3.7^ and 2 of ααα^anti4.2^, from the sample bank of the Medical Genetics Department in Southern Medical University (Guangzhou, China) (Table 1, Figure 1A). The clinical genomic samples had previously undergone MLPA to identify complex SVs in the α‐globin genes. Informed consent was obtained from all subjects before collecting the samples.

### 2.2 PCR Amplification

In this study, we used a set of primers similar to our previous study [5], but with some modification (Table 2, Figure 1B) to improve the production of amplicons ‐‐^*SEA*^ and to generate nine specific target sequencing templates including the allelic amplicons for α/β thalassemia deletion and special structure variations, as well as the whole‐gene sequence amplicons of *HBA2/1*, *HBG2/1*, *HBB*, and *HBD* for SNVs and SVs within these genes. These primers generate amplicons covering the most common and rare variants in the Chinese population, and it is possible to detect 912 types of SNVs and 56 types of SVs among these two gene clusters.

**TABLE 2:** Target amplicons and primers of two PCR reactions.

Target amplicons	Primer name	Sequence (5′‐3′)	Position	Concentration
HBA2	HBA2‐F	TCTGAGCTGAGGAAGCTGCATGTCCAC	chr16:169029–169,055	0.1 μM
	HBA2‐R	GCAGGAGGAACGGCTACCGAGGC	chr16:173607–173,629	0.018 μM
HBA1	HBA1‐F	CCCTCCTTGCACCGGCCCTTC	chr16:173658–173,678	0.018 μM
	HBA1‐R	GTGCCTGACTCGTAGCAGGTGTTTCTTC	chr16:179775–179,802	0.1 μM
HBA	HBA2‐F	TCTGAGCTGAGGAAGCTGCATGTCCAC	chr16: 169029–169,055	—
	HBA1‐R	GTGCCTGACTCGTAGCAGGTGTTTCTTC	chr16: 179775–179,802	—
THAI	THAI‐F	CAGCGCCACCCTCATGTCCATG	chr16: 142773–142,794	0.04 μM
	THAI‐R	CCTTGGATCTGCACCTCTGGGTAGGTTC	chr16: 183339–183,366	0.04 μM
HBDB	HBB‐F	CTGCACCTGCTGTGGCATCCATTC	chr11:5229660–5,229,683	0.03 μM
	HBDB‐R	CGTGGGAGAGAGGACAAGGACCACTTGAGAC	chr11:5224523–5,224,551	0.03 μM
HPFH	HBB‐F	CTGCACCTGCTGTGGCATCCATTC	ch11: 5229660–5,229,683	—
	HPFH‐R	GTGGCTGCTGAACTGAACTGTCCA	ch11: 5199099–5,199,122	0.016 μM
SEA	MSEA‐F	AGCGATCTGGGCTCTGTGTTCTC	chr16: 165255–165,277	0.03 μM
	MSEA‐R	AGCCCACGTTGTGTTCATGGC	chr16: 185889–185,909	0.03 μM
HBG	HBG‐F	CAGCCTCAGAGTTTTCCACATGCCCTTC	ch11:5251648–5,251,675	0.02 μM
	HBG‐R	GATGGGGCTGCTCTGCCTATGCAGTAGTC	ch11:5246661–5,246,686	0.02 μM
Chinese	HBG‐F	CAGCCTCAGAGTTTTCCACATGCCCTTC	chr11:5251648–5,251,675	—
	Chinese‐R	CCAGGCACCCATGACACAACTTCAAC	chr11:5169580–5,169,605	0.02 μM

Abbreviations: Chr, chromosome; F, forward; R, reverse.

We used two PCR reactions, and each reaction contained 3 μL gDNA (20 ng/μL) from both RMs and clinical samples. The PCR reaction 1 included the initial five pairs of primers listed in Table 2, whereas the PCR reaction two utilized the final three pairs of primers. Each PCR reaction was added with 4 μL 5 M Betaine (Sigma–Aldrich, St. Louis, MO), 2 μL DMSO (Sigma–Aldrich, St. Louis, MO), 7 μL 2.5 mM dNTPs, 10.6 μL PCR primer mixes in PCR reaction 1 and 6 μL PCR primer mixes in PCR reaction 2, 0.3 μL LA Taq polymerase, 5 μL 10 × LA PCR Buffer (Takara Bio Co. Ltd., Dalian, China) and 18.1 μL and 22.7 μL nuclease‐free water (NFW) in PCR reaction 1 and 2 respectively. PCR reaction conditions for optimal fragment amplification were at 95°C for 10 min (1 cycle); 95°C for 1 min, 68°C for 10 min (30 cycles); 72°C for 30 min (1 cycle), and hold at 4°C. The PCR products were purified with 0.5× volume (25 μL) Ampure XP beads (Beckman Coulter, Brea, CA), and DNA was eluted in 15 μL NFW, and then quantified with fluorometry (Qubit).

### 2.3 Library Preparation

The library preparation was performed by the Ligation Sequencing Kit (SQK‐LSK109; Oxford Nanopore Technologies, Oxford, United Kingdom). Pooled two PCR reaction products (200 ng from PCR reaction 1 and 100 ng from PCR reaction 2) into a new tube, and then end repair and dA‐tailing (NEBNext Ultra II End‐Repair/dA‐tailing Module) were performed by adding 3.5 μL Ultra II End‐Prep reaction buffer, 1.5 μL Ultra II End‐Prep enzyme mix, and NFW to 50 μL. The mixture was incubated at 20°C for 30 min and 65°C for 30 min. A 1× volume (30 μL) AMPure XP clean‐up was performed and the DNA was eluted in 13 μL NFW.

Native barcode ligation was performed by adding 11.5 μL purified products, 1.25 μL Native barcode (EXP‐NBD104 and EXP‐NBD114, Oxford Nanopore Technologies, Oxford, United Kingdom), 12 μL Blunt/TA Ligase Master Mix (NEB Blunt/TA Ligase Master Mix, M0367), and 0.5 μL Ligation buffer. The mixture was incubated at room temperature for 10 min. Then the barcode ligated DNA was cleaned up by adding 1× volume (25 μL) AMPure XP beads, and DNA was eluted in 15 μL NFW. Quantified 1 μL of barcoded DNA using a Qubit fluorometer.

Adapter Ligation was then performed by adding 32.5 μL purified barcoded DNA, 2.5 μL Adaptor Mix II (EXP‐NBD104 or EXP‐NBD114, Oxford Nanopore Technologies, Oxford, United Kingdom), 10 μL NEBNext Quick Ligation Reaction Buffer (NEBNext Quick Ligation Reaction Buffer, NEB B6058) and 5 μL Quick T4 DNA Ligase (Quick T4 DNA Ligase in NEBNext Quick Ligation Module (NEB E6056)), mixing gently and incubating at room temperature for 15 min. The adaptor‐ligated DNA was cleaned up by adding a 0.8× volume (40 μL) of AMPure XP beads, incubating for 5 min at room temperature, and resuspending the pellet twice in 200 μL LFB (SQK‐LSK109). The purified‐ligated DNA was eluted in 14 μL EB (SQK‐LSK109). A 1‐μL aliquot was quantified by fluorometry (Qubit).

### 2.4 Nanopore Sequencing and Data Analysis

MinION Mk1B sequencing was performed as per the manufacturer's guidelines using R9.4 flow cells (FLO‐MIN106, ONT). MinION sequencing was controlled using Oxford Nanopore Technologies MinKNOW software. We pooled 12 μL libraries, 37.5 μL SQB, and 25.5 μL LB together and loaded a total of 75 μL mixture on the flow cells. Before loading the library, we checked the flow cell to confirm the number of active pores, and the sequencing runs had a duration of 5 h.

To effectively detect the thalassemia alleles, an automated workflow was designed to evaluate the sequencing data from the MinION instrument. The sequencing depth more than 200× were required, and we also used fastp (version 0.21.0) [24] to remove the low‐quality reads from the raw data with the phred quality score (*Q* score) < 7. The raw reads were processed using Minimap2 (version 2.17‐r941) [25] and then sorted and indexed using SAMtools (version 1.7) [26, 27] to create a BAM file. The human genome database(hg38) was used for alignment. Since each sample was amplified with pairs of primers, the subset of BAM files was extracted according to primers coordination via customed scripts for targeted SVs identification. While the SNVs and insertions‐deletions (indels) were detected using Deepvariant version 1.1.0 [28] in Docker environment. The alignments of variants and the reference alleles were then shown in Integrative Genomics Viewer version 2.12.3.

The concordance of nanopore sequencing was determined based on its ability to accurately detect true positive and negative variants across all samples. Precision was ascertained by conducting 10 replicate tests with eight selected samples, encompassing one α‐globin SNV, two α‐globin deletions, two β‐globin SNVs, one β‐globin SV, and one triplication. All replicate tests were initiated from input genomic DNA. The limit of detection (LOD) for nanopore sequencing was assessed by conducting triplicate tests on the lowest detectable reference at concentrations of 10, 5, and 3 ng/μL.

## 3 Result

Across 172 tests (including 72 for precision evaluation and 64 for LOD assessment), sequencing was carried out on MinION flow cells. The average depths of three main target amplicons‐HBA, HBB and HBG were 6246×, 7538× and 10,657×, respectively. And the average read length across the flow cells was measured 4800 base pairs(bp). Additionally, 99.99% of the reads displayed at least one alignment to the human reference (GRCh38) and the target mapped ratio was 93.21%.

### 3.1 The Genotype Results by Nanopore Sequencing

We detected three wild type genotypes, which aligned with the RMs genotypes. A total of 19 distinct types of SNVs were identified, comprising 3 *HBA* and 16 *HBB* point mutations (Table 3), all of which showed complete concordance (100%) with the genotypes of the RMs in China. Additionally, we identified 11 deletion samples with six distinct deletion variants, including four of *HBA* and two of *HBB* (Table 3). Furthermore, all four additional α‐globin triplications were successfully detected.

**TABLE 3:** The results of SNVs and SVs by nanopore sequencing.

Sample name	Genetic variants	Sequencing results	
		Detected SNVs	Detected SVs
YSH2015‐0002	Hb E/β^N^	*HBB*: c.79G>A	
YSH2015‐0003	−28 (A>G)/β^N^	*HBB*: c.−78A>G	
YSH2015‐0004	IVS‐II‐654 (C>T)/β^N^ ‐‐^SEA^/αα	*HBB*: c.316‐197C>T	‐‐^SEA^
YSH2015‐0005	‐‐^SEA^/αα		‐‐^SEA^
YSH2015‐0006	−α^3.7^/‐‐^SEA^		−α^3.7^ ‐‐^SEA^
YSH2015‐0007	−α^3.7^/αα		−α^3.7^
YSH2015‐0008	IVS‐I‐1 (G>T)/β^N^	*HBB*: c.92+1G>T	
YSH2015‐0010	Codons 71/72 (+A)/β^N^	*HBB*: c.216_217insA/T	
YSH2015‐0011	Codon 17 (A>T)/β^N^	*HBB*: c.52A>T	
YSH2015‐0012	α^QS^ α/αα	*HBA2*: c.377T>C	
YSH2015‐0013	5′UTR; +43 to +40 (−AAAC)/β^N^	*HBB*: c.‐11_‐8delAAAC	
YSH2015‐0014	Codon 43 (G>T)/β^N^	*HBB*: c.130G>T	
YSH2015‐0015	Initiation Codon (ATG>AGG)/β^N^	*HBB*: c.2T>G	
YSH2015‐0017	Codon 37 (G>A)/β^N^	*HBB*: c.113G>A	
YSH2015‐0018	Codons 41/42 (−TTCT)/β^N^	*HBB*: c.126_129delCTTT	
YSH2015‐0019	−α^4.2^/αα		−α^4.2^
YSH2015‐0020	Codons 27/28 (+C)/β^N^	*HBB*: c.84_85insC	
YSH2015‐0021	α^CS^α/αα	*HBA2*: c.427T>C	
YSH2015‐0022	α^WS^α/αα	*HBA2*: c.369C>G	
YSH2015‐0023	−50 G>A/β^N^	*HBB*: c.‐100G>A	
YSH2015‐0024	−29 (A>G)/β^N^	*HBB*: c.−79A>G	
YSH2015‐0026	^G^γ(^A^γδβ)^0^/β^N^		^G^γ(^A^γδβ)^0^
YSH2015‐0027	Codons 41/42 (−TTCT)/^G^γ(^A^γδβ)^0^	*HBB*: c.126_129delCTTT	^G^γ(^A^γδβ)^0^
YSH2015‐0028	Codons 14/15 (+G)/β^N^	*HBB*: c.45_46insG	
YSH2015‐0029	−90 (C>T)/β^N^	*HBB*: c.−140C>T	
YSH2015‐0030	IVS‐II‐654 (C>T)/β^N^ −α^4.2^/−α^4.2^	*HBB*: c.316‐197C>T	−α^4.2^
YSH2015‐0031	IVS‐II‐654(C>T)/IVS‐II‐654 (C>T) ‐‐^SEA^/αα	*HBB*: c.316‐197C>T	‐‐^SEA^
YSH2015‐0032	SEA‐HPFH/β^N^		SEA‐HPFH
YSH2015‐0033	‐‐^THAI^/αα		‐‐^THAI^
S1	ααα^anti3.7^/αα		ααα^anti3.7^
S2	ααα^anti3.7^/αα		ααα^anti3.7^
S3	ααα^anti4.2^/αα		ααα^anti4.2^
S4	ααα^anti4.2^/αα		ααα^anti4.2^

Abbreviations: SNVs, single nucleotide variations; SVs, structural variations.

**FIGURE 2:**
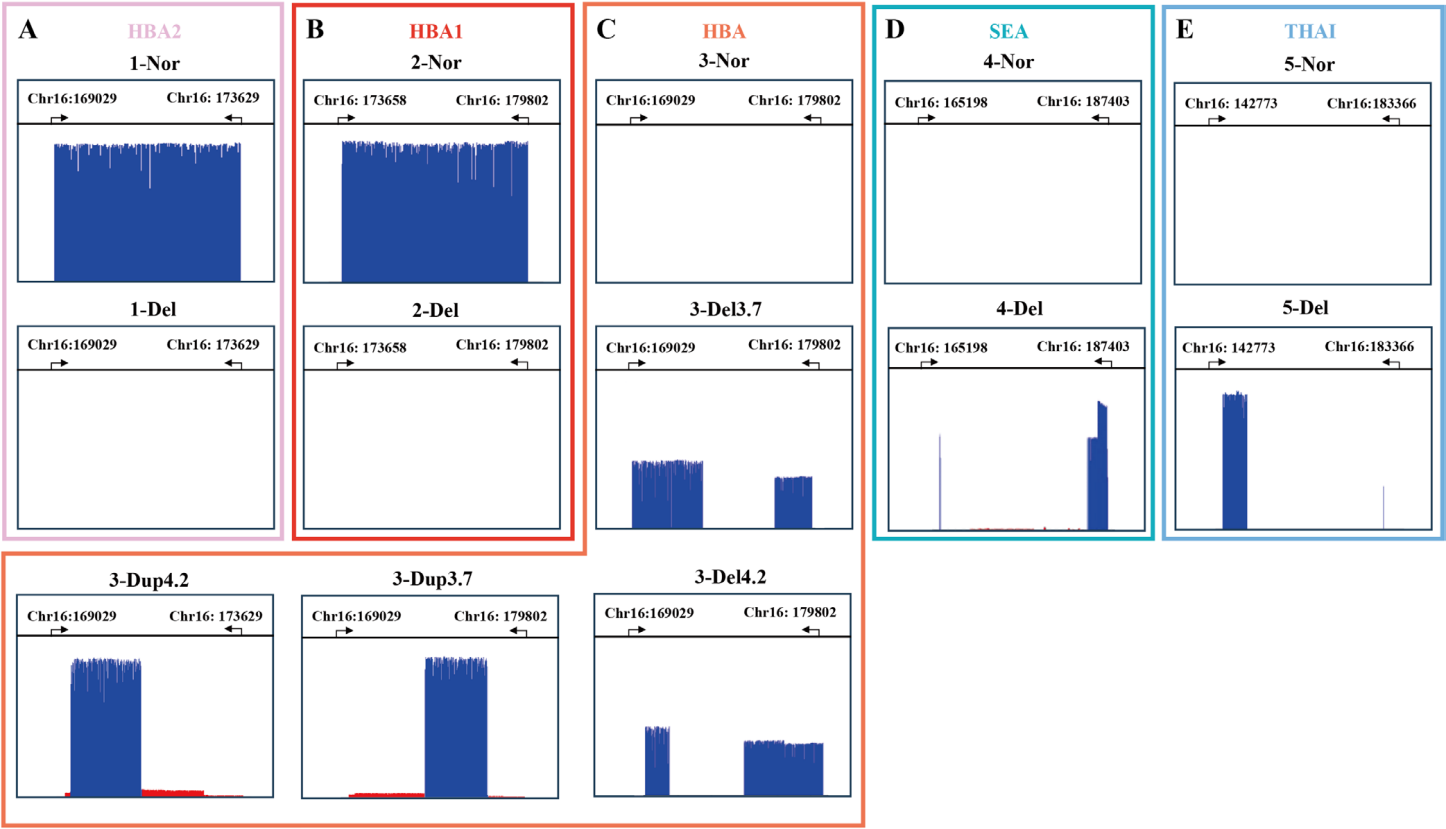
Interpretation of SVs of α‐globin gene. The blue band means sequencing depth of the target amplicons within the α‐globin gene. A, B, C, D and E represent the amplicons for HBA2, HBA1, HBA, SEA, and THAI, respectively. If A to E exhibit the sequencing depth as in “Nor”, then the sample is a normal genotype. If A, B, D and E present the “Del”, and C present “Del3.7” or “Del4.2”, then we could identify the sample as −α^3.7^ or −α^4.2^. Similarly, If A, B present as “Del”, C present as “Nor”, while D or E present as “Del”, then we could interpret the sample as ‐‐^SEA^ or ‐‐^THAI^ deletions. If A, B, D and E present the “Del”, and C present “Dup3.7” or “Dup4.2”, then we could identify the sample ααα^anti3.7^ or ααα^anti4.2^ triplications. Nor, normal; Del, deletion; Dup, duplication.

As depicted in Figure 2, A to E represent the amplicons for HBA2, HBA1, HBA, SEA, and THAI, respectively. In samples with a normal genotype, only the HBA2 and HBA1 amplicons were detectable, with lengths of 4.6 and 6.1 kb, respectively. The HBA, SEA, and THAI amplicons were not detectable due to their excessively long fragment sizes. For deletions of −α^3.7^ or −α^4.2^, the HBA2 and HBA1 amplicons were absent, while the HBA amplicon revealed a gap‐spanning product of approximately 7.0 or 6.6 kb, indicating deletions of 3.7 or 4.2 kb, respectively. Similarly, in the case of ‐‐^SEA^ or ‐‐^THAI^ deletions, the HBA2 and HBA1 amplicons were undetectable, but the SEA or THAI amplicons yielded a product of 2.8 or 7.2 kb, corresponding to deletions of 19.4 or 33.5 kb, respectively. In cases of ααα^anti3.7^ or ααα^anti4.2^ triplications, both HBA2 and HBA1 amplicons were detected, along with an additional PCR product of 3.7 or 4.2 kb observed in the HBA amplicon.

**FIGURE 3:**
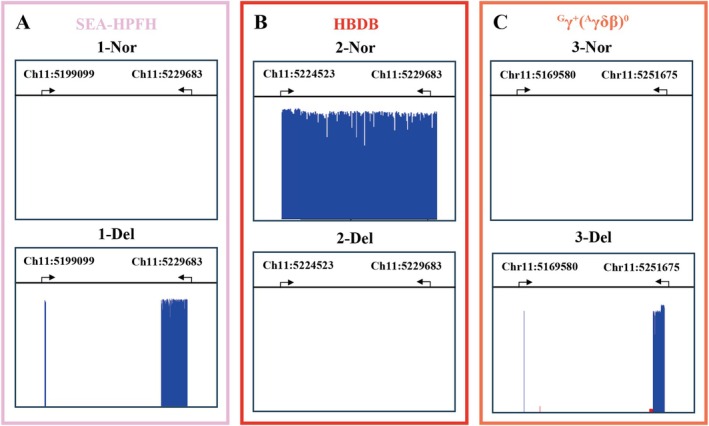
Interpretation of SVs of β‐globin gene. The blue bands signify sequencing depth of the target amplicons within the β‐globin genes. A, B, and C correspond to the amplicons of SEA‐HPFH, HBDB, and ^G^γ(^A^γδβ)^0^, respectively. If A, B, and C present “Nor”, then we could interpret the sample as normal genotypes. If B present “Del”, and A or C present “Del”, then we could identify the sample as SEA‐HPFH or ^G^γ(^A^γδβ)^0^ deletions. Nor, normal; Del, deletion.

In Figure 3, A, B, and C correspond to the amplicons of SEA‐HPFH, HBDB, and ^G^γ(^A^γδβ)^0^, respectively. For normal genotypes, the SEA‐HPFH and ^G^γ(^A^γδβ)^0^ amplicons were undetectable due to their excessive lengths, while only the HBDB amplicon was identified with a product length of 5.1 kb. For deletions of SEA‐HPFH or ^G^γ(^A^γδβ)^0^, the HBDB amplicon showed no detectable reads, whereas the SEA‐HPFH or ^G^γ(^A^γδβ)^0^ amplicons revealed a gap‐spanning product of 3.3 kb or 3.2 kb, corresponding to deletions of 27 or 78.9 kb, respectively. These amplification profiles allow for the differentiation of various *HBA* and *HBB* SVs.

### 3.2 Performance Evaluation

All genotype results by nanopore sequencing were consistent with the RMs and additional clinical samples. The concordance of nanopore sequencing in identifying thalassemia variants achieved 100%. Results from 10 replicates demonstrated complete concordance with the known genotype, confirming a precision of 100% (Table S1). The National Institutes for Food and Drug Control (NIFDC) in China recommend that the LOD should be less than 10 ng/μL. In this study, stable detection was achieved for selected samples at concentrations of 10, 5, and 3 ng/μL (Table S2), indicating that the LOD of nanopore sequencing meets the NIFDC's requirements.

## 4 Discussion

Accurate genetic diagnosis of thalassemia variants is supportive for a reproductive choice which is essential for alleviating the burden on health services. The molecular diagnostic potential of nanopore sequencing for identifying thalassemia mutations, by utilizing genetic DNA RMs or clinical samples, is promising. The high accuracy, concordance, and precision, along with a low LOD, of nanopore sequencing in detecting thalassemia mutations promise significant enhancements in screening and diagnosing carriers of thalassemia.

Traditional thalassemia diagnostic tests based on PCR in China [29] focus only on the most common variants associated with α and β thalassemia major. While these methods allow for the simultaneous detection of multiple thalassemia mutations in a single reaction, enabling screening for a relatively broader range of mutations, they can be cumbersome and potentially less effective. Additionally, these methods are limited to known mutations, posing challenges in detecting large deletions or insertions that may contribute to thalassemia. In contrast, nanopore sequencing generates long reads that usually span the breakpoints of the SVs [30]. This technology provides detailed information on the breakpoint of SVs, including precise length and nucleotide resolution, thereby facilitating the identification of complex thalassemia SVs including large deletions and triplications.

Moreover, nanopore sequencing offers several advantages for the genetic testing of thalassemia mutations. First, the technology generates long read lengths, providing a more comprehensive view of genomic regions [5], which enables the identification of additional regions of interest alongside the confirmation of known variants. For example, nanopore sequencing can uncover the plasticity of complex genes such as the *MHC* and *KIR* systems, allowing for high‐resolution characterization of genes, alleles, and phased haplotypes [19]. Second, as a form of un‐manipulated DNA sequencing, nanopore sequencing minimizes the introduction of biases and maintains the integrity of the original sequences, enabling researchers to focus on the regions of interest or capture underrepresented variants. Third, nanopore sequencing provides real‐time data generation, allowing for immediate base calling and sequencing results, significantly reducing the time required to generate actionable results. Lastly, nanopore sequencing exhibits flexibility, as it requires no additional assays or materials [31, 32]. Collectively, these attributes render nanopore sequencing a versatile and powerful tool applicable across various domains, including genomics, diagnostics, and beyond.

Recent studies have demonstrated that TGS is advantageous for detecting thalassemia variants, as the long amplicons span the full length of thalassemia alleles [5, 15, 18, 33]. Liang et al. [33] compared the PacBio single‐molecule‐real‐time (SMRT) sequencing platform with conventional thalassemia diagnostic methods and reported that their novel approach successfully identified all variants without any false negative results. Similarly, Luo et al. [15] demonstrated the effectiveness of the SMRT method in diagnosing thalassemia with rare deletions or complex SVs. Our previous study [5] established a nanopore sequencing method for detecting hemoglobinopathy variants, and in the current study, we further optimized the primer sets to enhance efficiency. Our findings consistently demonstrated high accuracy in thalassemia genetic testing. Although there is a certain error rate associated with nanopore sequencing in identifying SNVs, the base error at specific SNV positions is approximately 1.58% in our analysis, and this error rate does not significantly affect the normal/variants allele ratio, thereby ensuring accurate genotype identification. Our results indicated that all SNVs, deletions, and triplications were accurately identified, demonstrating complete concordance (100%) with the genotypes of the RMs in China and the additional clinical samples, along with low LOD.

The RMs are essential for monitoring test performance and ensuring quality in the development of new genetic tests. Consequently, RMs that contain a more comprehensive array of genetic variants are of significant importance. In this study, we accurately identified all genotypes of the RMs and the additional two types of α‐triplications using nanopore sequencing. Of note, the prevalence of ααα^anti3.7^ and ααα^anti4.2^ is about 1% to 2% among the general population of southern China [18, 34, 35]. Furthermore, α‐globin triplications can contribute to mild to severe β‐thalassemia intermedia in carriers of heterozygous β^0^ or β^+^ variants. Thus, we recommend that the RMs include these specific variants to broaden the range of variant types included in performance evaluation.

Challenges remain in the process of long‐read amplification in our study. First, the high homology among globin genes, combined with the long‐range amplicons in our study, poses a risk of producing artificial PCR‐hybrids [36]. However, we did not observe any such hybrids, which may be attributed to the high specificity of our primers, the optimization of PCR reaction conditions, and the inclusion of dimethyl sulfoxide (DMSO) and betaine in the PCR reaction. Second, for complex SVs, such as inversions or insertions of repetitive‐rich elements, it can be challenging to detect such variations [36]. On the other hand, all samples in this study were from China, which raises questions about the applicability of this technology for detecting variants that may be more prevalent in other countries, as well as its effectiveness for thalassemia variant detection outside of China.

In this study, we developed a classification method for thalassemia mutations, enabling automated interpretation, visual representation, and identification of diverse mutation types. We evaluated the performance of nanopore sequencing in thalassemia variations identification by utilizing national genomic RMs and four α‐globin triplication samples. Our findings demonstrated that nanopore sequencing comprehensively detects a wide range of thalassemia variants, including both common and rare SNVs, deletions, and triplications in a single assay. This technology exhibits complete concordance, precision, and low LOD, which serves as a potential assay for thalassemia variants detection. Furthermore, its ability to detect diverse variations may pave the way for its application in other genetic disorders. The scalability and cost‐effectiveness of this technology suggest promising prospects for its utilization in the broader landscape of genetic diagnostics.

## Author Contributions

X.W., X.Y., and X.x.Y. conceived and designed the study. L.Z. and S.Q. collected and performed the experiments. X.W., X.Y., S.Q., W.H., and G.O. analyzed and interpreted the data. X.W. drafted and wrote the paper. X.W., X.Y., W.H., and G.O. participated in discussing and revising the paper. S.Q., F.Y., and X.x.Y. contributed to overall senior mentorship and guidance, and support to the project. All authors listed have contributed to the work and approved the final version.

## Ethics Statement

The study was approved by the Institutional Review Board (IRB) of the Internal Ethics Committee of Zhujiang Hospital, Southern Medical University (authorization number: 2023‐KY‐301‐01).

## Conflicts of Interest

The authors declare no conflicts of interest.

## Supporting information


Data S1.

## Data Availability

The datasets in this article are available in the Genome Sequence Archive for human, under the accession number PRJCA038473 at https://ngdc.cncb.ac.cn/gsa‐human.
