# Supplementary material for: Applying the National Genomic DNA Reference Materials to Evaluate the Performance of Nanopore Sequencing in Identifying Thalassemia Variants
Source: J Clin Lab Anal. 2025 May 20;39(11):e70044. doi: 10.1002/jcla.70044 (PMC12144573; doi:10.1002/jcla.70044)
Supplement: Supplementary file 1 — Data S1. [file JCLA-39-e70044-s001.docx]

Supplementary Table 1 Precision results of nanopore sequencing of 8 selected samples which replicated 10 times.

| **Selected samples** | **Nanopore sequencing results of ten replications** | | | | | | | | | |
| --- | --- | --- | --- | --- | --- | --- | --- | --- | --- | --- |
|  | **R1** | **R2** | **R3** | **R4** | **R5** | **R6** | **R7** | **R8** | **R9** | **R10** |
| YSH2015-0002 | HBB: c.79G>A | HBB: c.79G>A | HBB: c.79G>A | HBB: c.79G>A | HBB: c.79G>A | HBB: c.79G>A | HBB: c.79G>A | HBB: c.79G>A | HBB: c.79G>A | HBB: c.79G>A |
| YSH2015-0007 | -α^3.7^ | -α^3.7^ | -α^3.7^ | -α^3.7^ | -α^3.7^ | -α^3.7^ | -α^3.7^ | -α^3.7^ | -α^3.7^ | -α^3.7^ |
| YSH2015-0012 | HBA2: c.369C>G | HBA2: c.369C>G | HBA2: c.369C>G | HBA2: c.369C>G | HBA2: c.369C>G | HBA2: c.369C>G | HBA2: c.369C>G | HBA2: c.369C>G | HBA2: c.369C>G | HBA2: c.369C>G |
| YSH2015-0015 | HBB: c.2T>G | HBB: c.2T>G | HBB: c.2T>G | HBB: c.2T>G | HBB: c.2T>G | HBB: c.2T>G | HBB: c.2T>G | HBB: c.2T>G | HBB: c.2T>G | HBB: c.2T>G |
| YSH2015-0026 | ^G^γ(^A^γδβ)^0^ | ^G^γ(^A^γδβ)^0^ | ^G^γ(^A^γδβ)^0^ | ^G^γ(^A^γδβ)^0^ | ^G^γ(^A^γδβ)^0^ | ^G^γ(^A^γδβ)^0^ | ^G^γ(^A^γδβ)^0^ | ^G^γ(^A^γδβ)^0^ | ^G^γ(^A^γδβ)^0^ | ^G^γ(^A^γδβ)^0^ |
| YSH2015-0032 | SEA-HPFH | SEA-HPFH | SEA-HPFH | SEA-HPFH | SEA-HPFH | SEA-HPFH | SEA-HPFH | SEA-HPFH | SEA-HPFH | SEA-HPFH |
| S1 | ααα^anti3.7^ | ααα^anti3.7^ | ααα^anti3.7^ | ααα^anti3.7^ | ααα^anti3.7^ | ααα^anti3.7^ | ααα^anti3.7^ | ααα^anti3.7^ | ααα^anti3.7^ | ααα^anti3.7^ |
| S4 | ααα^anti4.2^ | ααα^anti4.2^ | ααα^anti4.2^ | ααα^anti4.2^ | ααα^anti4.2^ | ααα^anti4.2^ | ααα^anti4.2^ | ααα^anti4.2^ | ααα^anti4.2^ | ααα^anti4.2^ |

Supplementary Table 2 LOD results of nanopore sequencing in thalassemia identification.

| **Samples name** | **Different dilution concentrations** | | | | | | | | | | | | | |
| --- | --- | --- | --- | --- | --- | --- | --- | --- | --- | --- | --- | --- | --- | --- |
|  |  | **10 ng/μL** | | |  |  | **5 ng/μL** | | |  |  | **3 ng/μL** | | |
|  | **R1** | | **R2** | **R3** | | **R1** | | **R2** | **R3** | | **R1** | | **R2** | **R3** |
| YSH2015-0001 | - | | - | - | | - | | - | - | | - | | - | - |
| YSH2015-0004 | HBB: c.316-197C>T  --^SEA^ | | HBB: c.316-197C>T  --^SEA^ | HBB: c.316-197C>T  --^SEA^ | | HBB: c.316-197C>T  --^SEA^ | | HBB: c.316-197C>T  --^SEA^ | HBB: c.316-197C>T  --^SEA^ | | HBB: c.316-197C>T  --^SEA^ | | HBB: c.316-197C>T  --^SEA^ | HBB: c.316-197C>T  --^SEA^ |
| YSH2015-0011 | HBB: c.52A>T | | HBB: c.52A>T | HBB: c.52A>T | | HBB: c.52A>T | | HBB: c.52A>T | HBB: c.52A>T | | HBB: c.52A>T | | HBB: c.52A>T | HBB: c.52A>T |
| YSH2015-0015 | HBB: c.2T>G | | HBB: c.2T>G | HBB: c.2T>G | | HBB: c.2T>G | | HBB: c.2T>G | HBB: c.2T>G | | HBB: c.2T>G | | HBB: c.2T>G | HBB: c.2T>G |
| YSH2015-0019 | -α^4.2^ | | -α^4.2^ | -α^4.2^ | | -α^4.2^ | | -α^4.2^ | -α^4.2^ | | -α^4.2^ | | -α^4.2^ | -α^4.2^ |
| YSH2015-0023 | HBB: c.-100G>A | | HBB: c.-100G>A | HBB: c.-100G>A | | HBB: c.-100G>A | | HBB: c.-100G>A | HBB: c.-100G>A | | HBB: c.-100G>A | | HBB: c.-100G>A | HBB: c.-100G>A |
| YSH2015-0030 | -α^4.2^  HBB: c.316-197C>T | | -α^4.2^  HBB: c.316-197C>T | -α^4.2^  HBB: c.316-197C>T | | -α^4.2^  HBB: c.316-197C>T | | -α^4.2^  HBB: c.316-197C>T | -α^4.2^  HBB: c.316-197C>T | | -α^4.2^  HBB: c.316-197C>T | | -α^4.2^  HBB: c.316-197C>T | -α^4.2^  HBB: c.316-197C>T |
| S3 | ααα^anti4.2^ | | ααα^anti4.2^ | ααα^anti4.2^ | | ααα^anti4.2^ | | ααα^anti4.2^ | ααα^anti4.2^ | | ααα^anti4.2^ | | ααα^anti4.2^ | ααα^anti4.2^ |

These samples were diluted to three different concentrations, and repeated twice. LOD, limit of detection.
